# Supplementary figures and images for: Effects of different educational interventions on cervical cancer knowledge and human papillomavirus vaccination uptake among young women in Japan: Preliminary results of a cluster randomized controlled trial
Source: PLoS One. 2025 Jan 7;20(1):e0311588. doi: 10.1371/journal.pone.0311588 (PMC11706404; doi:10.1371/journal.pone.0311588)

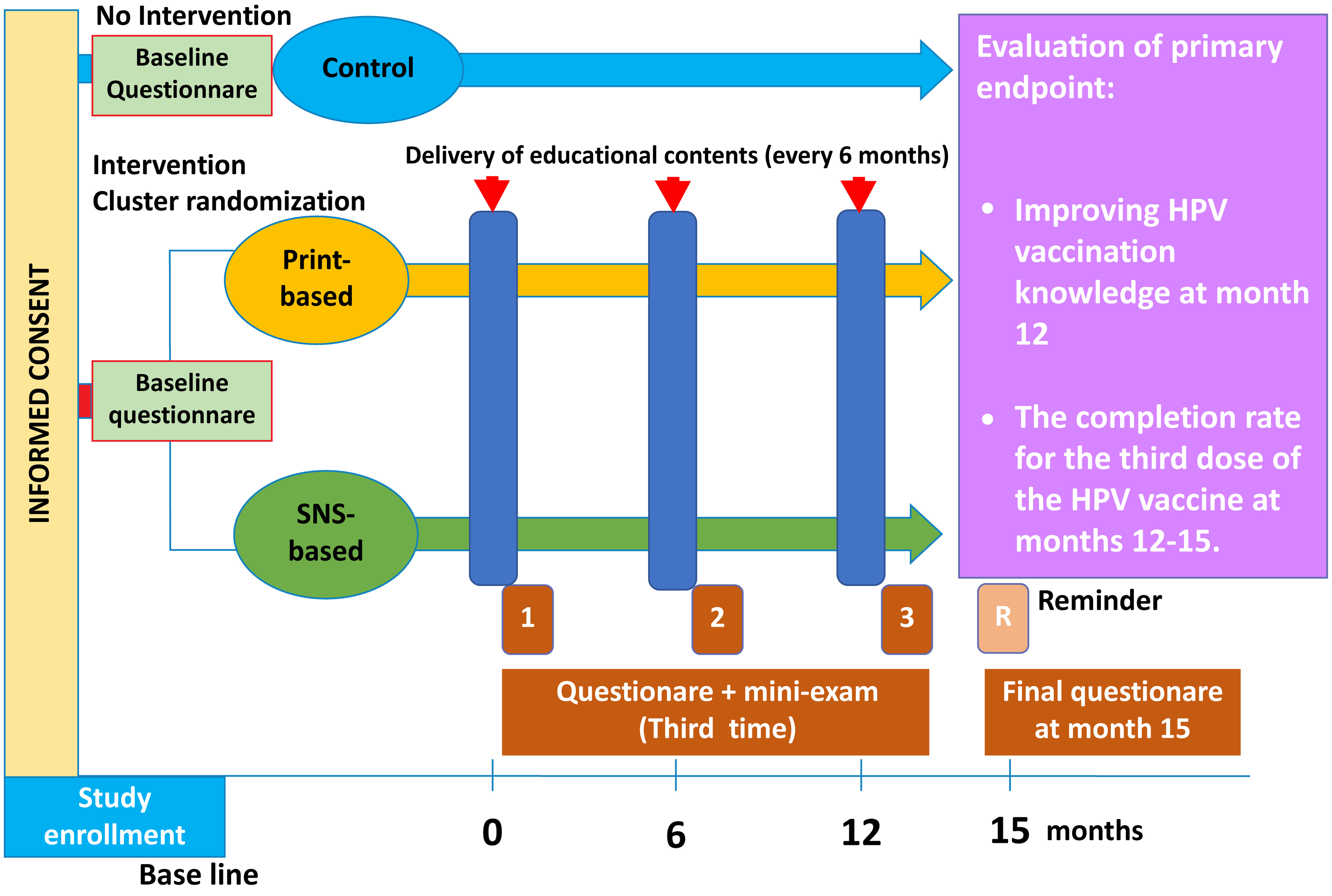

Supplement: S1 Fig — (TIF) [file pone.0311588.s001.tif]

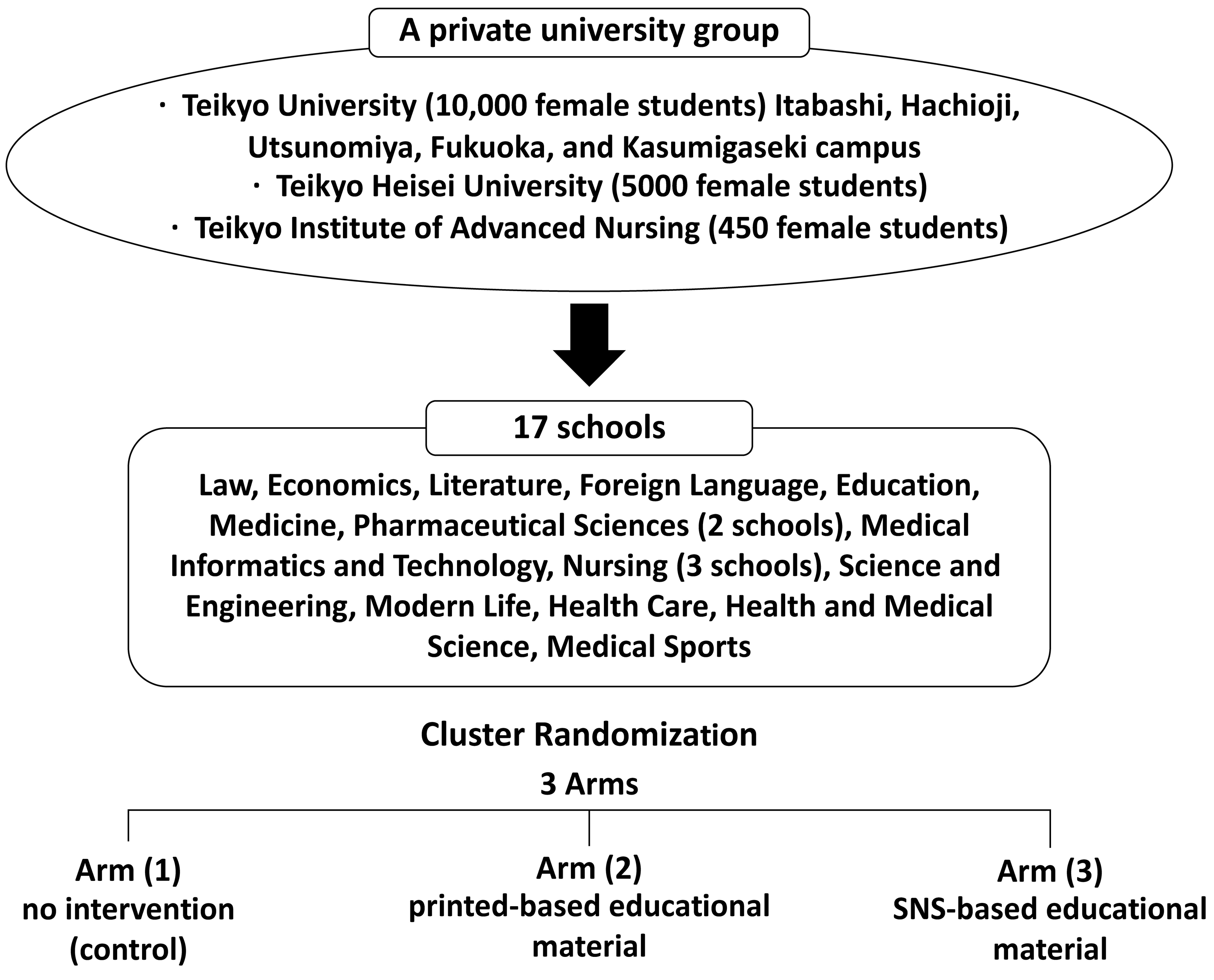

Supplement: S2 Fig — (TIF) [file pone.0311588.s002.tif]

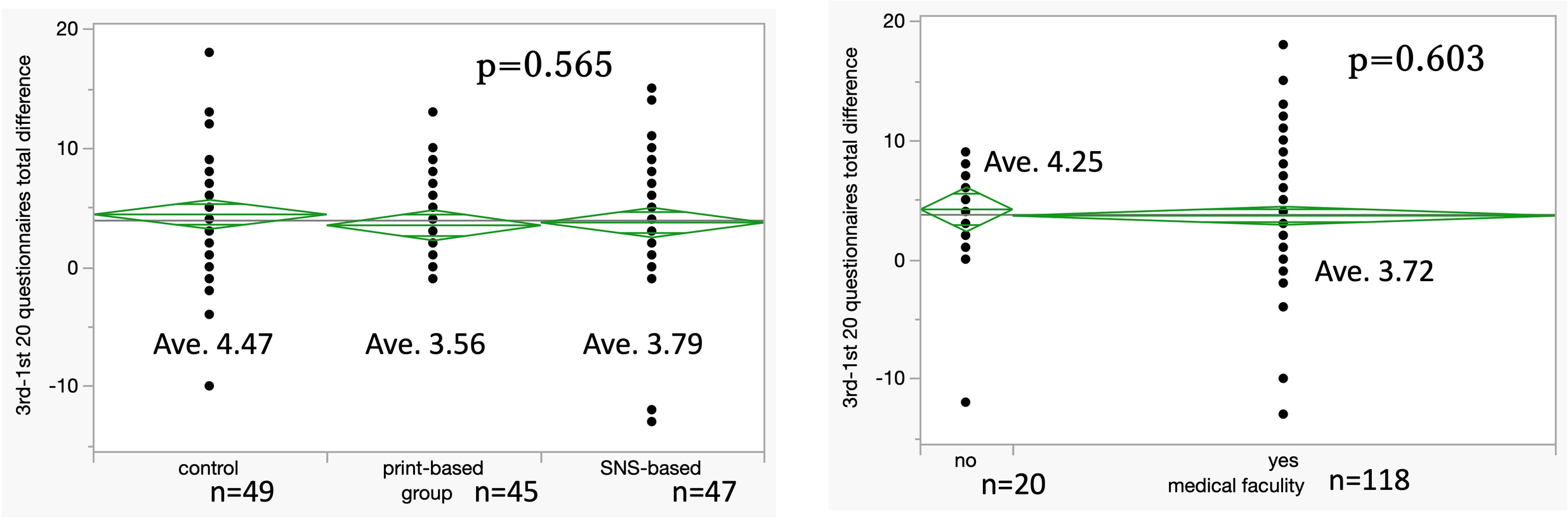

Supplement: S3 Fig — No significant differences were found among the three education groups, whereas non-medical students faculty tend to show greater improvements than did those in a medical faculty. (TIF) [file pone.0311588.s003.tif]
