# Supplementary material for: Effects of different educational interventions on cervical cancer knowledge and human papillomavirus vaccination uptake among young women in Japan: Preliminary results of a cluster randomized controlled trial
Source: PLoS One. 2025 Jan 7;20(1):e0311588. doi: 10.1371/journal.pone.0311588 (PMC11706404; doi:10.1371/journal.pone.0311588)
Supplement: S1 Method — (PDF) [file pone.0311588.s004.pdf]

Supplemental Methods.

Questions about your current life

Q1 Please indicate your affiliation ________

Q2 Please indicate your age _______

Q3 Do you have any family members in the medical profession?

Q3-1 If yes, who is it? (Please circle all that apply)

1. father 2. mother 3. grandfather 4. grandmother

5. aunt 6. uncle 7. cousin 8. brother 9. other (free answer:)

Q3-2→ Do you live with your family?

1. yes 2. no

Q4 Do you smoke?

1. yes 2. no

Q5 Do you exercise voluntarily?

Q5-1 If yes

1. one day a week 2. two or three days a week 3. every day

Q6 Are you conscious of eating a well-balanced diet on a regular basis?

1. yes 2. no

Q7 Have you ever visited an obstetrician or gynecologist?

1. yes 2. no

Q7-1 If you answered "No," please explain why.

1. I have no chance to see a doctor

2. I have symptoms that concern me, but I am reluctant to see a doctor (Reason:)

3. I don't know where to see a doctor

4. other (free answer:)

Q8 What symptoms or illnesses have you had? (Please circle all that apply)

Irregular menstruation, abnormal menstrual flow, headache, fatigue, malaise, lack of concentration, visual field disorder, visual disturbance, hearing impairment, vertigo, insomnia, sleep disorder, skin disease, hyperventilation, memory loss, poor calculation ability, poor memory for Kanji characters, involuntary movements, gait disorder, limb weakness, headache, fatigue, lack of concentration, Involuntary movement, gait disturbance, limb weakness, allergy, asthma, diabetes, epilepsy, syncope, arrhythmia, hypertension, Others (free answers:)

Q8-1 How old were you when (since) you had the above symptoms or diseases? ________

Q9 Have you ever received any of the routine immunizations (BCG, MR vaccine, etc.)

provided under Japanese law?

1. yes 2. no 3. I have no idea. (Reason:)

Q10 Please circle the frequency regarding your history of vaccination against human papillomavirus (HPV) vaccine (also called cervical cancer prevention vaccine).

1. never vaccinated 2. one time 3. two times 4. three times

Q10-1 Questions for those who have received less than 3 doses of HPV vaccine (Those who chose 1, 2, or 3 in Q10). Please select the reason why you did not take the HPV vaccine. (Please circle all that apply.)

1. because I am afraid of the side effects of the vaccine.
2. because it costs money.
3. because my parents don't recommend it.
4. because my friends didn’t vaccinate.
5. because I don't think I will get infected.
6. because I haven't had sexual intercourse yet.
7. because I don't know where to get the vaccine.
8. because I think it is enough to have a checkup.
9. because I was a child at the time and didn't know anything about it.
10. because my school teacher didn’t recommend it.

(Free answer column: ________ ______________________________)

Q10-2 Questions for those who have received at least one HPV vaccine (those who selected 2, 3, or 4 in Q10)

Please tell us the age at which you received the HPV vaccine respectively. If you do not remember at all, please circle at the end.

1st:______ 2nd:______ 3rd: ______ No idea
