# Supplementary material for: Effects of different educational interventions on cervical cancer knowledge and human papillomavirus vaccination uptake among young women in Japan: Preliminary results of a cluster randomized controlled trial
Source: PLoS One. 2025 Jan 7;20(1):e0311588. doi: 10.1371/journal.pone.0311588 (PMC11706404; doi:10.1371/journal.pone.0311588)
Supplement: S1 Table — (PDF) [file pone.0311588.s005.pdf]

Supplemental Table S1. Questionnaire about knowledge of cervical cancer and the human papillomavirus vaccine

Please put a circle in either "I know" or "I did not know" about cervical cancer and the human papillomavirus vaccine.

	Items	I know	I did not know
(1)	Cervical cancer is an infectious disease caused by the human papillomavirus (HPV).		
(2)	There are more than 100 types of HPV and 14 types of HPVs that cause cervical cancer (high-risk HPV).		
(3)	Persistent infection with high-risk HPV causes cervical cancer.		
(4)	HPV is ubiquitous and common virus that can be transmitted to the uterus through even a single sexual activity.		
(5)	Over 80% of women who have ever had sexually transmitted infection will also experience HPV infection with age.		
(6)	HPV can be transmitted to not only women but also men.		
(7)	Cervical cancer is the most common cancer among women in their 20s and 30s.		
(8)	About 3000 patients pass away from cervical cancer every year in our country.		
(9)	There is possible delayed detection even if you have annual check-ups for cervical cancer.		
(10)	Even if cervical cancer is detected at an early stage, removal of the uterus is necessary.		
(11)	Even if an abnormality is detected in the stage before cervical cancer, you need to remove a part of the uterus, which may cause premature delivery.		
(12)	There is an HPV vaccine that can prevent cervical cancer.		
(13)	There is significant evidence that the HPV vaccine		

	can prevent cervical cancer.		
(14)	The HPV vaccine is more effective in preventing cervical cancer when given before infection with HPV (before sexual intercourse).		
(15)	If you have been vaccinated, you need to receive regular checkups for early detection of cancer because there is a possibility that you may be infected by HPV that cannot be prevented.		
(16)	It would be best to undergo a “catch-up vaccination”		
(17)	The adverse reactions to the HPV vaccine drew media attention 5 years ago in our country.		
(18)	No evidence or cause of adverse reactions to HPV vaccines in Japan has been found.		
(19)	There is scientific evidence on the efficiency and safety of the HPV vaccine.		
(20)	The Japan Society of Obstetrics and Gynecology strongly urges the resumption of active recommendation of HPV vaccine.		
