# Supplementary material for: Effects of different educational interventions on cervical cancer knowledge and human papillomavirus vaccination uptake among young women in Japan: Preliminary results of a cluster randomized controlled trial
Source: PLoS One. 2025 Jan 7;20(1):e0311588. doi: 10.1371/journal.pone.0311588 (PMC11706404; doi:10.1371/journal.pone.0311588)
Supplement: S2 Table — (PDF) [file pone.0311588.s006.pdf]

Supplemental Table S2. Communicative and Critical Health Literacy Scale (CCHL)

➢ Each item is rated on a 5-point scale, ranging from 1 (very easy) to 5 (very difficult).
➢ The scale score is calculated as the mean score for all five items. (theoretical range: 1–5)
➢ Items 1) - 3): Communicative health literacy, Items 4), 5): Critical health literacy

Q. Do you think you can find and utilize the health and medical information related to HPV vaccine and cervical cancer screening by yourself if you need? Please circle one number against each statement.
Please circle one number from "very easy" to "very difficult" that best describes your ability.

Items		Very easy	Slightly easy	Intermediate	Slightly difficult	Very difficult
(1)	I can collect information related to the human papillomavirus (HPV) vaccine and cervical cancer screening from various sources, such as newspapers, books, television, and the Internet.	1	2	3	4	5
(2)	I can extract the information you are looking for from a large selection of information related to the HPV vaccine and cervical cancer screening.	1	2	3	4	5
(3)	I can understand and communicate the obtained information related to the HPV vaccine and cervical cancer screening to others	1	2	3	4	5
(4)	I can judge the credibility of the information related to the HPV vaccine and cervical cancer screening.	1	2	3	4	5
(5)	I can make decisions about plans and actions for improving your health based on information related to the HPV vaccine and cervical cancer screening.	1	2	3	4	5
