# Supplementary material for: Effects of different educational interventions on cervical cancer knowledge and human papillomavirus vaccination uptake among young women in Japan: Preliminary results of a cluster randomized controlled trial
Source: PLoS One. 2025 Jan 7;20(1):e0311588. doi: 10.1371/journal.pone.0311588 (PMC11706404; doi:10.1371/journal.pone.0311588)
Supplement: S3 Table — (PDF) [file pone.0311588.s007.pdf]

Supplemental Table S3. Baseline characteristics of the female students who participated in the study from three universities in Japan, 2019–2023 (N = 188: number of participants)

	N	%
**Age (years)**		
18	7	3.7
19	23	12.2
20	37	19.7
21	54	28.7
22	32	17.0
23	16	8.5
24	11	5.9
25	4	3.9
26	3	1.6
**Affiliation**		
Pharmaceutical	36	20.7
Medicine	45	23.9
Nursing	50	27.1
Literature	15	8.0
Economics	5	2.7
Education	4	2.1
Law	3	1.6
Foreign language	3	1.6
Science and Technology	1	0.5
Medical Technology	28	14.9
**Medical professional in the family**		
Yes	72	38.3
No	116	61.7
**Living with parents**	37	19.7
**Smoking habit**	3	1.6
**Awareness regarding the importance of having a balanced diet**	127	67.6
**Exercising voluntarily**		
None	114	60.6
Once a week	29	15.4
Twice or three times per week	37	19.7
Every day	5	2.66
**History of consultation with Obstetrics and Gynecology specialists**	37	19.7
**Routine vaccinations conducted in accordance with Japanese law**		
	154	81.9
**HPV vaccination history**		
None	107	56.9
Once	18	9.6
Twice	14	7.4
Three times	48	25.5

HPV, human papillomavirus
