# Supplementary material for: Effects of different educational interventions on cervical cancer knowledge and human papillomavirus vaccination uptake among young women in Japan: Preliminary results of a cluster randomized controlled trial
Source: PLoS One. 2025 Jan 7;20(1):e0311588. doi: 10.1371/journal.pone.0311588 (PMC11706404; doi:10.1371/journal.pone.0311588)
Supplement: S4 Table — (PDF) [file pone.0311588.s008.pdf]

Supplemental Table S4. Factors related to “high” health literacy scores in the third survey

Factors	Comparison	OR (95% CI) for “High health literacy score”	p-value
Group (SNS-based)	vs. control	1.09 (0.31, 2.75)	0.888
Group (print-based)	vs. control	0.55 (0.15, 2.11)	0.391
Medical faculty	vs. non-Medical faculty	1.22 (0.22, 6.8)	0.816
Medical professional in the family	vs. none	0.81 (0.28, 2.38)	0.701
Smoked/Smoking	vs. never	4.67 (0.12, 185.5)	0.412
Exercise	vs. no	2.25 (0.76, 6.7)	0.146
Care about diet	vs. no	0.29 (0.09, 0.89)	0.031
Gyn visit	vs. no	1.34 (0.48, 3.69)	0.575
Routine vaccination	vs. no	0.36 (0.029, 2.26)	0.220
HPV vaccine	vs. never	2.36 (0.58, 9.58)	0.231
Completed three doses of HPV vaccine	vs. no	0.62 (0.14, 2.81)	0.533
1st CCHL scale score	high vs. low	5.77 (1.26, 26.43)	0.024
2nd CCHL scale score	high vs. low	7.93 (2.59, 24.3)	< 0.001

SNS, social networking service; CCHL, Communicative and Critical Health Literacy Scale; HPV, human papillomavirus ; OR, odds ratio; CI, confidence interval

Logistic regression analysis was used to estimate the adjusted ORs and 95% CIs for CCHL scale scoring (response variable: 1 = CCHL “high” in the 3rd survey, 0 = “low”).
