# Supplementary material for: Effects of different educational interventions on cervical cancer knowledge and human papillomavirus vaccination uptake among young women in Japan: Preliminary results of a cluster randomized controlled trial
Source: PLoS One. 2025 Jan 7;20(1):e0311588. doi: 10.1371/journal.pone.0311588 (PMC11706404; doi:10.1371/journal.pone.0311588)
Supplement: S5 Table — (PDF) [file pone.0311588.s009.pdf]

Supplemental Table S5. Distribution of knowledge regarding cervical cancer items and rate of the answer “I know” in the first, second, and third rounds (N = 141)

	*N1st	%	*N2nd	%	*N3rd	%
1) Cervical cancer is an infectious disease caused by the HPV.	117	83.0	128	90.8	132	93.6
2) There are more than 100 types of HPV and 14 types of HPVs that cause cervical cancer (high-risk HPV).	33	23.4	57	40.4	62	44.0
3) Persistent infection with high-risk HPV causes cervical cancer.	78	55.3	95	67.4	102	72.3
4) HPV is ubiquitous and common virus that can be transmitted to the uterus through even a single sexual activity.	103	73.0	126	89.4	135	95.7
5) Over 80% of women who have ever had sexually transmitted infection will also experience HPV infection with age.	49	34.8	64	45.4	78	55.3
6) HPV can be transmitted not only to women but also to men.	83	58.9	102	72.3	120	85.1
7) Cervical cancer is the most common cancer among women in their 20s and 30s.	82	58.2	100	70.9	107	75.9
8) About 3,000 patients pass away from cervical cancer every year in our country.	36	25.5	61	43.3	66	46.8
9) There is possible delayed detection even if you have annual checkups for cervical cancer.	67	47.5	93	66.0	101	71.6
10) Even if cervical cancer is detected at an early stage, removal of the uterus is necessary.	70	49.6	93	66.0	103	73.0
11) Even if an abnormality is detected in the stage before cervical cancer, you need to remove a part of the uterus, which may cause premature delivery.	53	37.6	81	57.4	104	73.8
12) There is an HPV vaccine that can prevent cervical cancer.	131	92.9	132	93.6	138	97.9
13) There is significant evidence that the HPV vaccine can prevent cervical cancer.	126	89.4	129	91.5	134	95.0
14) The HPV vaccine is more effective in preventing cervical cancer when	108	76.6	119	84.4	125	88.7

given before infection with HPV (before sexual intercourse).						
15) If you have been vaccinated, you need to receive regular checkups for early detection of cancer because there is a possibility that you may be infected by HPV that cannot be prevented.	98	69.5	112	79.4	120	85.1
16) It would be best to undergo a “catch-up vaccination”	46	32.6	71	50.4	102	72.3
17) The adverse reactions to the HPV vaccine drew media attention 5 years ago in our country.	114	80.9	121	84.8	128	90.8
18) No evidence or cause of adverse reactions to HPV vaccines in Japan has been found.	69	48.9	94	66.7	108	76.6
19) There is scientific evidence on the efficiency and safety of the HPV vaccine.	84	59.6	102	72.3	124	87.9
20) The Japan Society of Obstetrics and Gynecology strongly urges the resumption of active recommendation of HPV vaccine.	100	70.9	111	78.7	129	91.5

HPV, human papillomavirus

*The number of participants for each information.

Items No. 2 and 8: less than half of the participants had knowledge at the 1st instance, and this proportion remained at less than half at the 3rd instance. Items No. 3, 5, 9, 10, 11, 16, and 18: less than half of the participants had knowledge at the 1st instance; this proportion increased to more than half at the 3rd instance. Items No. 1, 4, 6, 7, 12, 13, 14, 15, 17, 19, and 20: more than half of the participants had knowledge at the 1st instance; this increased, especially for items No. 1, 4, 12, 13, 17, and 20, with knowledgeable participants accounting for over 90%.
