# Supplementary material for: Effects of different educational interventions on cervical cancer knowledge and human papillomavirus vaccination uptake among young women in Japan: Preliminary results of a cluster randomized controlled trial
Source: PLoS One. 2025 Jan 7;20(1):e0311588. doi: 10.1371/journal.pone.0311588 (PMC11706404; doi:10.1371/journal.pone.0311588)
Supplement: Attachment — Submitted filename: Reviewer comments for the article.docx [file pone.0311588.s010.docx]

Reviewer comments for the Manuscript Number PONE-D-24-35490

“Effects of different educational interventions on cervical cancer knowledge and human papilloma virus vaccination uptake among young women in Japan: preliminary results of a utcluster randomized controlled trial”

Kazunori Nagasaka et al.

**Abstract**

The abstract looks good and gives an overview of cervical cancer in Japan it could improve by providing more context about the adverse reactions that led to the suspension of the HPV vaccine could enhance understanding of vaccine hesitancy.

**Introduction**

This section is good. It can be improved by providing specific examples of how health literacy can be improved. Other areas the author needs to check are for instance;

- Line 70 Cooper et al. [24]... the reference style is not consistent with the other references. Ensure that references are consistent and clearly formatted to improve readability of the article.

**Materials and methods**

This section looks good but the authors will need to check and make correction on the following.

Line 86 a cluster, randomized, parallel-group trial - This is a bit confusing. did you mean parallel cluster randomized group trial?

Line 121 Clusters were defined as a faculty or department and an allocation. This statement is not clear, is there some information missing?

The authors indicate that Analysis of variance with Scheffé test was performed for continuous variables, and the Chi-square test ….. Was the Scheffé technique utilised in the analysis? If they did were the results presented in the writeud?

189  performed for categorical variables in the subgroup analysis.

Line 148 -149 A score of ≤3 was considered “highly health literate” because this represented the number of participants in the two groups. this statement is not clear, what is the connection of the literacy score and the number of participants?

Line 179 logistic regression mixed-effect-Is this, ok? I guess you wanted to say mixed-effect logistic model

On line 193 the authors state that, “these scores were summed, a lower total score indicated that the respondents were more knowledgeable ,. The researchers may need to reverse the score coding since most readers will associate high score to success. In this case it is in the opposite direction

Line 264- 265, the alternative hypothesis is not clear. Was the alternative hypothesis one sided since the authors state that hat the proportion of participants with high health literacy in Groups 2 and 3 would be higher than that in Group 1

line 365…, for the third questionnaire, ….do the authors mean the third survey here or what is the third questionnaire?

On line 381 and 383 the authors use the abbreviations MHLW which is not clearly defined.
